# Magnetic Field Characteristics of Multiple Niobium Three-dimensional Nano-bridge Junctions in Parallel

**DOI:** 10.1038/s41598-019-46425-z

**Published:** 2019-07-09

**Authors:** Xiaohan Chen, Lei Chen, Yue Wang, Long Wu, Xiaoyu Liu, Linxian Ma, Zhen Wang

**Affiliations:** 1 Center for Excellence in Superconducting Electronics (CENSE), Shanghai Institute of Microsystem and Information Technology (SIMIT), Chinese Academy of Sciences (CAS), Shanghai, 200050 China; 2 University of Chinese Academy of Science, Beijing, 100049 China; 3 Shanghai Tech University, Shanghai, 200031 China

**Keywords:** Superconducting devices, Surface patterning

## Abstract

The superconducting device of multiple Josephson junctions in arrays has increasingly attracted interest in both applications and fundamental research. The challenge of array integration and scaling is a wide concern. The present study investigated superconducting devices of multiple niobium three-dimensional nano-bridge junctions (3D-NBJs) in parallel. We fabricated evenly and unevenly spaced devices of three to six 3D-NBJs in parallel. We measured the critical current as a function of the magnetic field and voltage to magnetic field transfer function of each device. The derivative of voltage with respect to the magnetic field at the sensitive point increased linearly with the number of junctions. A maximal derivative of 97.3 V/T was achieved by our device with six unevenly spaced junctions in parallel. Furthermore, we carried out numerical simulations on devices of three and four junctions in parallel using the current–phase relation of a single 3D-NBJ. The CPR was determined by comparing the measured and simulated magnetic flux modulations of nano-SQUID. Qualitative agreement between the numerical simulation and experimental measurement suggests that it is possible to use 3D-NBJs to build SQUID arrays or SQIFs with high integration density.

## Introduction

Superconducting quantum interference devices (SQUIDs) comprising two Josephson junctions (JJs) in parallel are well-known sensitive magnetic sensors^1–4^. Recently, greater numbers of JJs have been connected into arrays, such as SQUID arrays and superconducting quantum interference filters (SQIFs), to produce more sensitive magnetometers^5–7^, absolute field magnetometers^8–10^, low-noise amplifiers^11–13^, and near-quantum-limit radio-frequency antennas^14–16^. Theoretically, the function of the transfer from the magnetic field signal to voltage improves linearly with the number of JJs^17–19^. The integration density of the JJ array therefore plays an important role. For instance, the length of an array must be less than an eighth of the wavelength to produce radio-frequency antennas^20^.

The niobium (Nb) three-dimensional nano-bridge junctions (3D-NBJs) that we developed previously exemplify miniaturization towards the nano-SQUID, which increases spin sensitivity^21^. The 3D-NBJs also have an advantageous non-hysteresis current–voltage curve with a relatively large voltage step (~0.5 mV) at the critical current^21,22^. Therefore, by using of these 3D-NBJs, not only same number of junctions will occupy less area, but also a larger field-to-voltage transfer ratio can be obtained. However, unlike the case for conventional tri-layer junctions, the physical model of 3D-NBJs remains unclear. The resistance as a function of the measured temperature indicates that the Josephson effect of 3D-NBJs may originate from quantum phase-slip (QPS) centers^21–24^. Meanwhile, a QPS-junction array in parallel has been suggested to be an ideal test bed for the superconductor–insulator phase transition with the ability to be tuned by magnetic frustrations^25,26^. It would therefore be intriguing to study 3D-NBJ arrays from both application and physics points of view.

This paper presents superconducting devices of three to six 3D-NBJs connected in parallel with even and uneven spacing. The critical currents of these devices were measured as a function of the magnetic field. We also characterized the magnetic-field-to-voltage transfer function of each device. The derivative of voltage with respect to the magnetic field at the sensitive point increased with the number of junctions. To further clarify the behavior of 3D-NBJs in parallel, we then determined the current-phase relation (CPR) of a single 3D-NBJ by comparison of the measured and simulated magnetic flux modulations of a nano-SQUID. On the basis of the CPR, we carried out numerical simulations on three and four junctions in parallel. Qualitative agreement between the numerical simulation and experimental measurement suggests that it is possible to use 3D-NBJs to build SQUID arrays or SQIFs with high integration density.

## Results and Discussion

**Figure 1:**
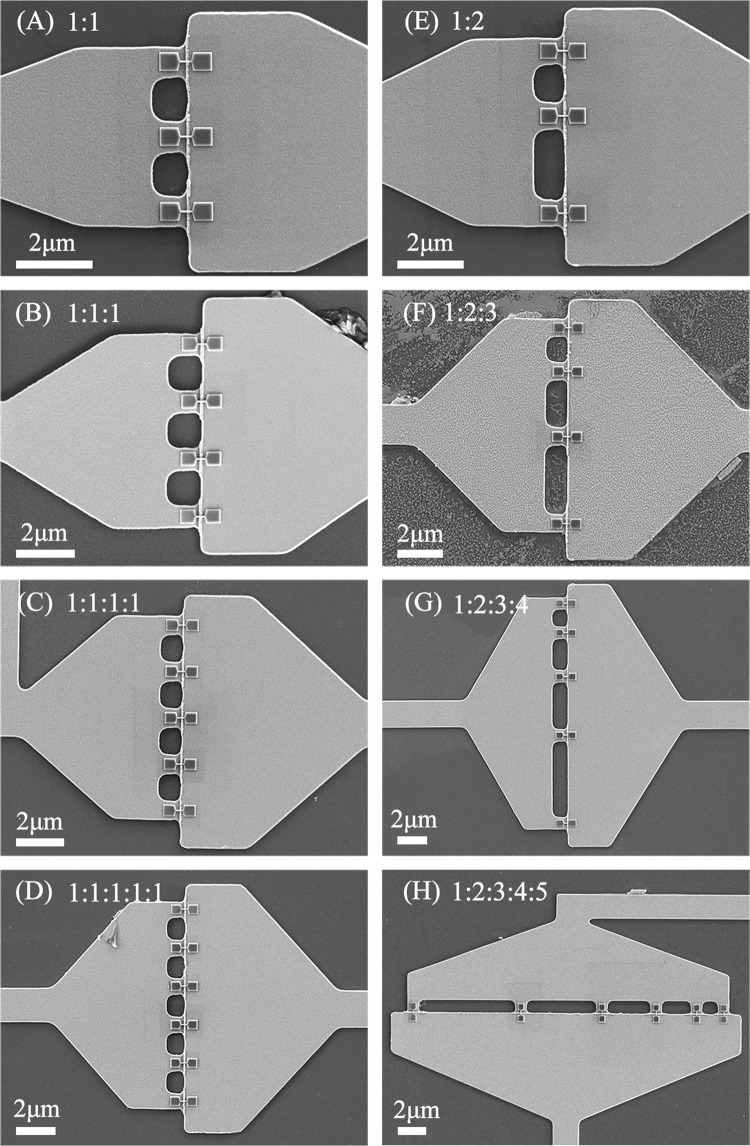
SEM images of eight devices of 3D nano-bridge junctions in parallel with (**A**–**D**) three to six evenly spaced 3D-NBJs and (**E**–**H**) three to six unevenly spaced 3D-NBJs.

Figure 1 shows that we fabricated eight devices of 3D NBJs in parallel (namely Dev. A–H) adopting a fabrication method that we previously developed^21,22^. Devices A–D and E–H have three to six junctions with even and uneven spacing, respectively. The uneven spacings of Dev. E, F, G, and H have ratios of 1:2, 1:2:3, 1:2:3:4, and 1:2:3:4:5, respectively. Their banks are designed to taper with relatively large areas to allow current to be distributed into all junctions uniformly.

**Figure 2:**
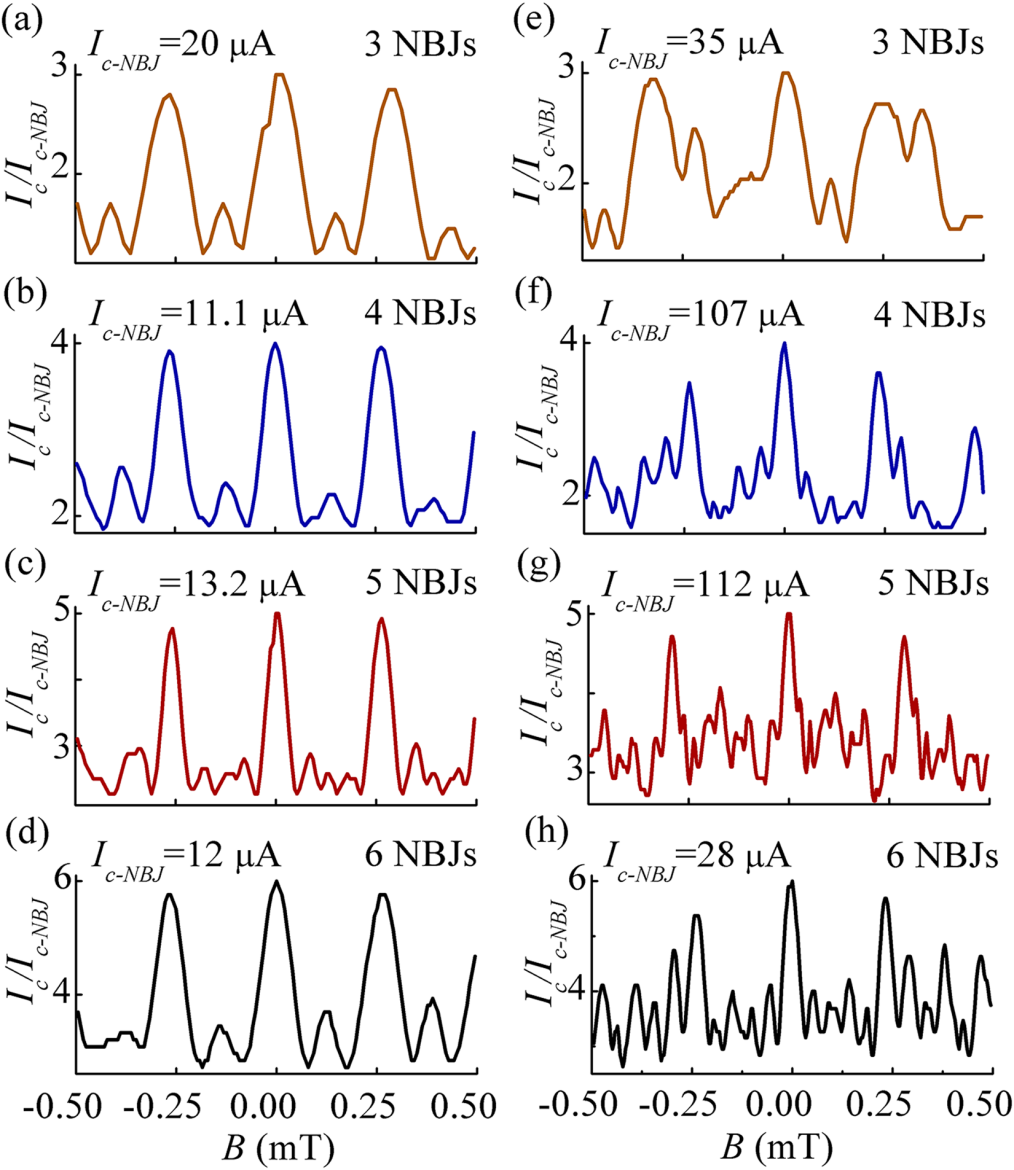
(**a**–**h**) Critical current *I*_*c*_ as a function of the applied magnetic field *B* of Dev. A–H. The critical current *I*_*c*_ of each device is normalized to the critical current of a single 3D-NBJ *I*_*c-NBJ*_.

Figure 2(a–h) respectively shows the critical current *I*_*c*_ as a function of the applied magnetic field *B* measured for Dev. A–H. All measurements were made at 4.2 K in liquid helium. For a clear comparison, we normalized the critical current *I*_*c*_ of each device to the critical current of a single 3D NBJ *I*_*c-NBJ*_ at zero magnetic field. Figure 2(a–d) shows that the evenly spaced devices bear similar periodic magnetic-field modulations. The modulation depth Δ*I*_*c*_/*I*_*c-max*_ is around 53.9–61.7%, which is similar to that for nano-SQUIDs made from 3D-NBJs. Compared with the case of a nano-SQUID, the periodic main peaks are narrower for multiple evenly spaced NBJs in parallel. Figure 2(e–h) shows that the unevenly spaced devices have much less obvious periodic modulation than evenly spaced devices. The main peaks are much narrower. There is more enhanced small fluctuation at the bottom of the modulation of unevenly spaced devices. The critical currents of individual NBJs *I*_*c-NBJ*_ in these devices differ from device to device. We examined each device under a scanning electron microscope (SEM) after electric measurement and found that none of the junctions appeared to have physical damage. We therefore believe that the variation in *I*_*c-NBJ*_ comes from local roughness of the insulating groove under the nano-bridge as we discussed in reference^22^. A higher spread in *I*_*c-max*_ of unevenly spaced devices is observed with respect to the evenly ones, because they are more distant from each other. However, it is reasonable to presume that *I*_*c-NBJ*_ is relatively uniform, at least within the scope of a single device; there would otherwise be skewed peaks instead of symmetric peaks in the magnetic-field modulation.

**Figure 3:**
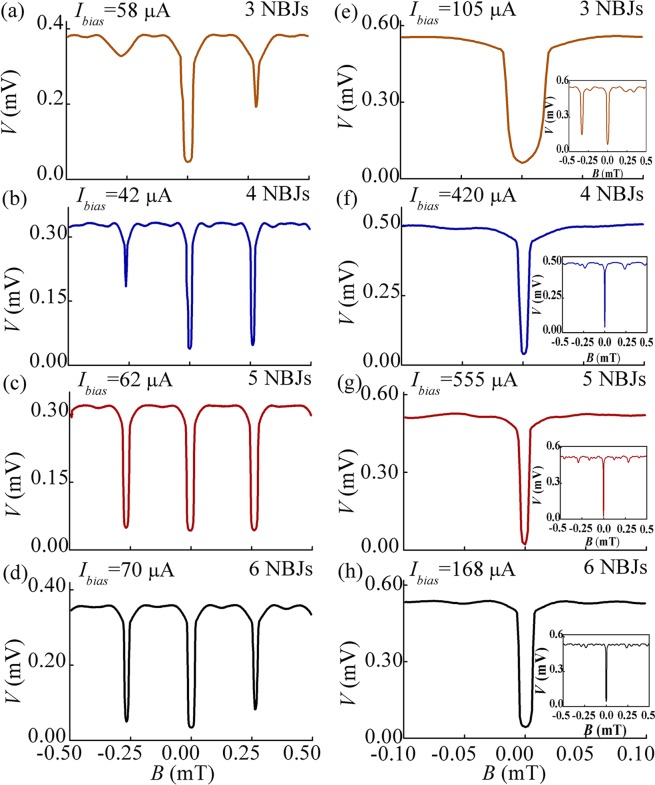
(**a**–**h**) *B*-to-*V* transfer functions of Dev. A–H, respectively. The insets of (**e**–**h**) show the wider magnetic field range.

We also measured the magnetic-field-to-voltage (*B*-to-*V*) transfer function of Dev. A–H at a fixed bias current *I*_*bias*_, as respectively shown in Fig. 3(a–h). The value of *I*_*bias*_ of each device is listed in Table 1 and selected to obtained the maximal d*V*/d*B*. As expected, the *B*-to-*V* transfer functions of evenly spaced Dev. A–D are periodic as shown in Fig. 3(a–d). Meanwhile, the unevenly spaced Dev. E–H have an obvious central dip around a zero magnetic field. The insets of Fig. 3(e–h) show the wider magnetic field range. Except for Dev. E having three NBJs, both side dips are suppressed, and the *B*-to-*V* transfer function has a behavior similar to that of a SQIF. The generated voltage signal approaches 0.5 mV on average, which is an advantage in the application of magnetic sensing.

**Table 1:** Corresponding values of the zero-field critical current *I*_*c-max*_, critical current of a single 3D-NBJ *I*_*c-NBJ*_, bias current *I*_*bias*_ used to measure the *V*-to-*B* transfer function, and the screen parameter 2*LI*_*c-NBJ*_/*Ф*_*0*_ for each device.

Dev.#	evenly spaced				unevenly spaced			
	A	B	C	D	E	F	G	H
*I*_*c-max*_	74 μA	66 μA	44.5 μA	60 μA	168 μA	560 μA	429 μA	106 μA
*I*_*c-NBJ*_	12 μA	13.2 μA	11.1 μA	20 μA	28 μA	112 μA	107 μA	35 μA
*I*_*bias*_	70 μA	62 μA	42 μA	58 μA	168 μA	555 μA	420 μA	105 μA
*2LI*_*c-NBJ*_/*Ф*_*0*_	0.267	0.293	0.247	0.444	0.622	2.489	2.378	0.778

**Figure 4:**
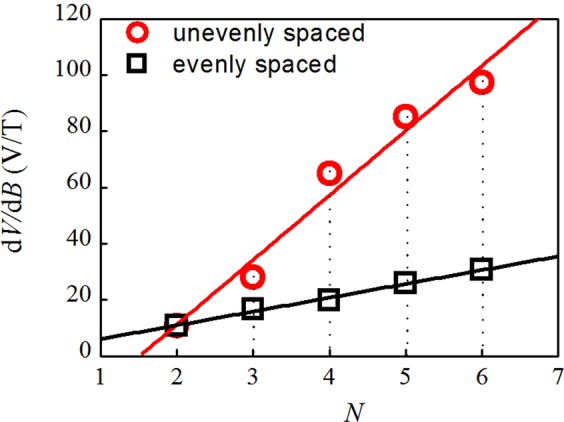
Maximum value of d*V*/d*B* as a function of *N*. The black squares and red circles respectively represent evenly spaced and unevenly spaced devices.

From the *B*-to-*V* transfer function in Fig. 3, we can calculate the derivative d*V*/d*B* and plot the maximum value as a function of *N* in Fig. 4, where *N* is the number of NBJs. The open black squares and red circles respectively represent the evenly and unevenly spaced devices. Both sets of points can be fitted using a straight line, which indicates that d*V*/d*B* of the devices of 3D-NBJs in parallel increases with *N* and that d*V*/d*B* of unevenly spaced devices is obvious greater than that of evenly spaced devices with the same numbers of junctions. A maximal transfer function of 97.3 V/T was achieved by 6 unevenly spaced junctions in parallel.

**Figure 5:**
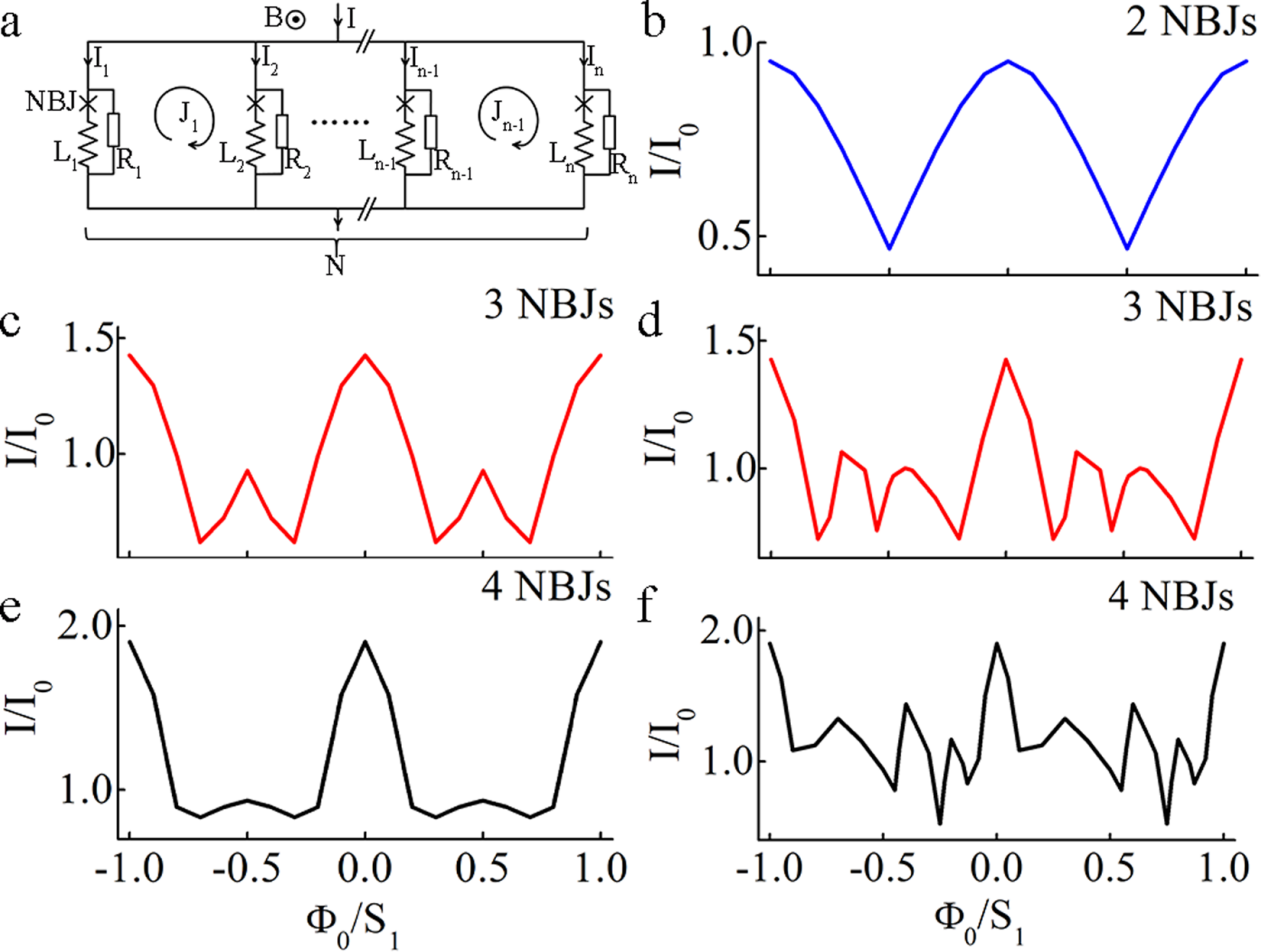
(**a**) Circuit diagram of N 3D-NBJs in parallel; (**b**) numerical simulation of the critical current as a function of the applied magnetic field of a device with two NBJs in parallel (SQUID); (c) numerical simulation of the critical current as a function of the applied magnetic field of a device with three NBJs in parallel spaced evenly and (**d**) spaced unevenly; (**e**) numerical simulation of the critical current as a function of the applied magnetic field of a device with four NBJs in parallel spaced evenly and (**f**) spaced unevenly.

Figure 5 is a plot of the simulated critical current as a function of the magnetic field of multiple 3D-NBJs in parallel with even and uneven spacing. The simulation is based on equations (1), (2) and (3) described in the method section. We here only ran the simulation for *N* = 3 and 4 owing to the limited resources of a personal computer. However, the simulation helps us understand the experimental results. Firstly, the magnetic modulation depth is the same as that of a nano-SQUID, which is determined by the screen parameter and inductance. Secondly, simulation results show a periodicity of *Ф*_*0*_/*S*_*1*_, which is consistent with the experimental data. Here *S*_*1*_ is the loop area for the smallest spacing. Lastly, the even spacing has modulation behavior more similar to that of a SQUID, but the main peaks are narrower than those of a SQUID. Unevenly spaced 3D-NBJs in parallel exhibit even narrower main peaks and the bottom floor of the modulation bears more fluctuations, which is consistent with experiments.

Our experimental and simulation results for devices of multiple 3D-NBJs in parallel therefore agree qualitatively. Albeit for a non-sinusoidal CPR, the multiple 3D-NBJs have magnetic field characteristics that are similar to those of conventional JJs and scale with the number of junctions. The devices have the advantage of a large non-hysteresis voltage signal around 0.5 mV. It is therefore promising to use 3D-NBJs in superconducting devices for array applications with an immediate increase in integration density. However, the uniformity of critical currents is a technical challenge that needs to be overcome beforehand. We are able to tune the critical current in the experiments by tuning the physical dimensions of the NBJs. It is also reasonable to presume that the NBJs are largely uniform within the small scope of our device as indicated by the symmetric main peaks of the magnetic field modulations. We believe that the ruggedness left by the lift-off process contributed to the fluctuations of the critical currents from device to device. This may be mitigated by introducing chemical–mechanical polishing before setting the NBJs. However, it is unknown whether there is a more fundamental parameter than the physical dimensions that determines the critical currents of NBJs. The exact physical model of the 3D-NBJ remains in the shadows and requires further investigation.

## Methods

### Device fabrication

Firstly, two layers of Nb film having thickness of 150 nm were deposited via direct-current magnetron sputtering on a silicon wafer. Both Nb layers were patterned adopting ultraviolet photolithography followed by CF_4_ reactive-ion etching. Before the second Nb layer was deposited, a 20-nm MgO layer was deposited to produce an 8-nm MgO layer on the sidewall of the first Nb layer. After lifting off, an 8-nm MgO insulating slit formed between the two 150-nm-thick Nb banks. Then, 12-nm-thick and 50-nm-wide Nb nano-bridge junctions were patterned across the insulating slit through electron-beam lithography.

### Estimation of the CPR

**Figure 6:**
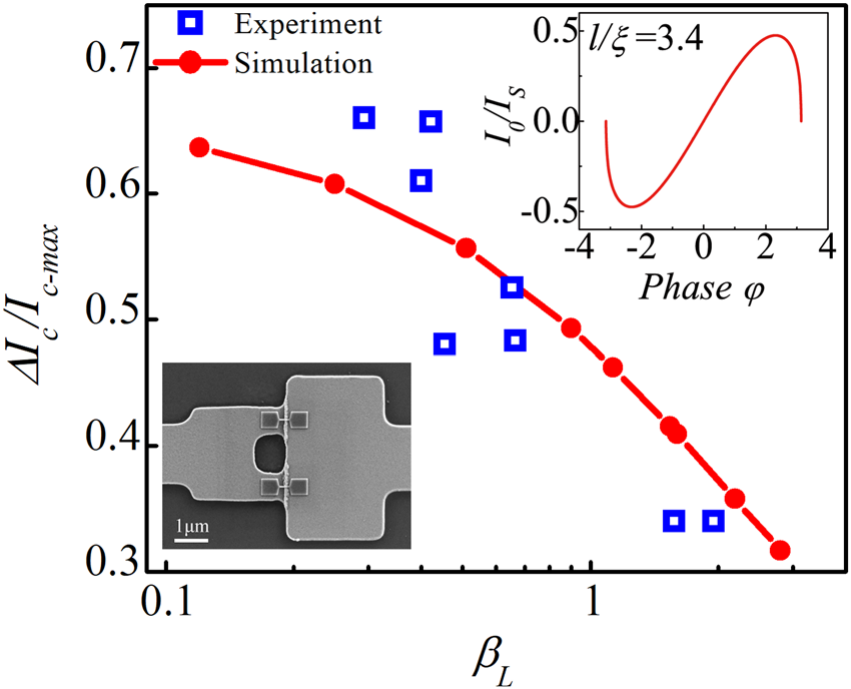
Magnetic flux modulation depth Δ*I*_*c*_/*I*_*c-max*_ as a function of the screen parameter *β*_*L*_. Blue squares and red dots are respectively measurements and simulation results. The top inset shows the CPR calculated using the model described in reference^28^ and the parameter *l*/*ξ* = 3.4. The bottom inset shows the SEM image of a SQUID made from 3D-NBJs.

Figure 6 presents measurements of eight nano-SQUIDs made from 3D-NBJs. The thickness and width of an NBJ were respectively 12 and 50 nm, as shown in the bottom inset of Fig. 6. The nano-SQUIDs were designed to be same and fabricated at the same time as the devices described above. The empty blue squares plots the magnetic flux modulation depth Δ*I*_*c*_/*I*_*c-max*_ as a function of the screen parameter *β*_*L*_ = 2*LI*_*c-NBJ*_/*Ф*_*0*_, where *L* and *I*_*c-NBJ*_ are respectively the loop inductance and critical current of a single NBJ and *Ф*_*0*_ is the flux quantum. We used a typical value of *L* = 23 pH as in reference^27^. For a simple comparison, we assume that all 3D-NBJs follows a typical constant CPR. Red dots are values of the modulation depths calculated using the CPR P_*l*/*ξ*_(*φ*) as shown in the upper inset of Fig. 6 instead of a sinusoid function for the SQUID model^28,29^. The CPR is calculated using the model in reference^28^ with *l*/*ξ* = 3.4, where *l* is the effective length of a 3D-NBJ and *ξ* is the superconducting coherence length of Nb. Qualitative agreement between the measurements and simulation results indicates that the typical CPR of our 3D-NBJs follows the function plotted in the inset of Fig. 6.

### Model for a 3D NBJ array in parallel

Adopting the CPR *P*_*l*/*ξ*_(*φ*) of a 3D-NBJ, we can use equations (1), (2) and (3) to describe the electrical transportation behavior of 3D-NBJs in parallel, which can be easily obtained by analogy to a SQUID model^28,29^. The *I*_*c*_–*B* modulation of a constant *N* 3D-NBJs in parallel can therefore be calculated. The even and uneven spacings of the junctions can be determined by *S*_*n*_, the effective area between neighboring junctions^30^. Here, *I*_*n*_, *L*_*n*_, and *J*_*n*_ are respectively the supercurrent across the 3D-NBJ, inductance of the 3D-NBJ, and circulating current. *I*_*0n*_ is the maximum critical current of the 3D-NBJ.

1 $$\{\begin{array}{rcl}{I}_{1}+{J}_{1} & = & {I}_{01}{P}_{l/\xi }({\phi }_{1})+\frac{1}{{R}_{1}}\frac{\hslash }{2e}\frac{d{\phi }_{1}}{dt}\\ {I}_{2}-{J}_{1}+{J}_{2} & = & {I}_{02}{P}_{l/\xi }({\phi }_{2})+\frac{1}{{R}_{2}}\frac{\hslash }{2e}\frac{d{\phi }_{2}}{dt}\\ \cdots  &  & \\ {I}_{n}-{J}_{n-1} & = & {I}_{0n}{P}_{l/\xi }({\phi }_{n})+\frac{1}{{R}_{n}}\frac{\hslash }{2e}\frac{d{\phi }_{n}}{dt}\end{array}$$

2 $$\{\begin{array}{rcl}{\phi }_{1}-{\phi }_{2} & = & \tfrac{2\pi }{{\varphi }_{0}}[B{S}_{1}-{L}_{1}({I}_{1}+{J}_{1})+{L}_{2}({I}_{2}-{J}_{1}+{J}_{2})]\\ {\phi }_{2}-{\phi }_{3} & = & \tfrac{2\pi }{{\varphi }_{0}}[B{S}_{2}-{L}_{2}({I}_{2}+{J}_{2}-{J}_{1})+{L}_{3}({I}_{3}+{J}_{3}-{J}_{2})]\\ \cdots  &  & \\ {\phi }_{(n-1)}-{\phi }_{n} & = & \tfrac{2\pi }{{\varphi }_{0}}[B{S}_{(n-1)}-{L}_{(n-1)}({I}_{(n-1)}+{J}_{(n-1)}-{J}_{(n-2)})+{L}_{n}({I}_{n}-{J}_{(n-1)})]\end{array}$$

3 $$\{\begin{array}{rcl}V & = & {V}_{1}+{L}_{1}\tfrac{d{J}_{1}}{dt}={V}_{2}+{L}_{2}\tfrac{d(\,-\,{J}_{1}+{J}_{2})}{dt}=\cdots ={V}_{(n-1)}+{L}_{(n-1)}\tfrac{d(\,-\,{J}_{(n-2)}+{J}_{(n-1)})}{dt}={V}_{n}+{L}_{n}\tfrac{d(\,-\,{J}_{n})}{dt}\\ \tfrac{d{\phi }_{1}}{dt} & = & \tfrac{2e}{\hslash }{V}_{1}\\ \cdots  &  & \\ \tfrac{d{\phi }_{n}}{dt} & = & \tfrac{2e}{\hslash }{V}_{n}\end{array}$$

## Conclusion

We studied superconducting devices made from multiple niobium 3D-NBJs in parallel. The 3D-NBJs ranged in number from three to six and were evenly and unevenly spaced. The critical current of parallel 3D-NBJs arrays as a function of the applied magnetic field and *B*-to-*V* transfer function was measured for each device. A maximal transfer function of 97.3-V/T was achieved by our device of 6 unevenly spaced junctions in parallel. Furthermore, we simulated the magnetic field modulation of three and four NBJs in parallel arrays using a typical CPR of our 3D-NBJs. The typical CPR was obtained by comparing experimental and simulated flux modulation depths of nano-SQUIDs with a fitting parameter *l*/*ξ* = 3.4. The measured and simulated magnetic field modulations were in qualitative agreement with each other. In addition, the measured d*V*/d*B* of devices of multiple 3D-NBJs has a linear scaling relation with respect to *N*. In principle, a larger transfer function can be achieved by connecting more 3D-NBJs in arrays whose size will be much smaller than the one made by other existing junctions technology. Therefore, the use of 3D-NBJs is a promising approach for improving the integration density of superconducting devices that require a large number of junctions.
